# AdapTree: Data-Driven Approach to Assessing Plant Stress Through the AI-Sensor Synergy

**DOI:** 10.3390/s25103149

**Published:** 2025-05-16

**Authors:** Divisha Garg, Harpreet Singh, Yosi Shacham-Diamand

**Affiliations:** 1 Department of Computer Science Engineering, Thapar Institute of Engineering and Technology, Patiala 147004, Punjab, India; divisha.bfcet@gmail.com; 2 School of Electrical Engineering, Tel-Aviv University, Tel Aviv 6997801, Israel; yosish@tauex.tau.ac.il; 3 Scojen Institute of Synthetic Biology, Reichman University, Herzliya 4610101, Israel

**Keywords:** impedance, plant sensors, plant stress, relative humidity, temperature, machine learning

## Abstract

This study investigates plant stress assessment by integrating advanced sensor technologies and Artificial Intelligence (AI). Multi-sensor data—including electrical impedance spectroscopy, temperature, and humidity—were used to capture plant physiological responses under environmental stress conditions. The key task addressed was the prediction of stress-related parameters using machine learning. A novel boosting-based ensemble method, AdapTree, combining AdaBoost and decision trees, was proposed to improve predictive accuracy and model interpretability. Experimental evaluation across multiple regression metrics demonstrated that AdapTree outperformed baseline models, achieving an R^2^ score of 0.993 for impedance magnitude prediction and 0.999 for both relative humidity (RH) and temperature, along with low root mean squared error (134.565 for impedance, 0.006966 for RH, and 0.0050099 for temperature) and mean absolute error values (22.789 for impedance; 1.51 × $10^{-5}$ for RH and 2.51 × $10^{-5}$ for temperature). These findings validate the reliability and effectiveness of the proposed AI-driven framework in accurately interpreting sensor data for plant stress detection. The approach offers a scalable, data-driven solution to enhance precision agriculture and agricultural sustainability. Furthermore, this method can be extended to monitor additional stress markers or applied across diverse plant species and field conditions, supporting future developments in intelligent crop monitoring systems.

## 1. Introduction

Plants are highly responsive to environmental conditions that significantly influence their physiological processes, growth, and productivity. Abiotic stressors such as temperature extremes, varying humidity levels, and fluctuating light intensities can disrupt essential functions like photosynthesis, transpiration, and nutrient uptake, ultimately reducing crop yield and quality. High temperatures may induce cellular damage and lower photosynthetic activity, while low temperatures can suppress metabolic reactions and hinder plant development [1,2]. Humidity extremes affect stomatal regulation and water balance, increasing the risk of desiccation or fungal infection [3]. Additionally, insufficient or excessive light disrupts energy capture and biomass accumulation, adversely impacting growth. These environmental stress factors pose critical challenges to sustainable agriculture.

The impact of environmental stress on plant health has been extensively studied, highlighting its effects on various physiological and biochemical processes. Traditional methods of stress detection—primarily visual inspection and manual physiological assessments—are labor-intensive, subjective, and lack the precision and scalability required for modern, large-scale agricultural practices [4,5]. In recent years, convergence of technology and agriculture has resulted in the development of modern diagnostic tools that enhance both the accuracy and efficiency of stress detection. Modern approaches to plant stress diagnosis include: (1) Thermal and multispectral imaging to detect water stress and nutrient deficiencies [6]. (2) Fluorescence sensors to monitor photosynthetic efficiency. (3) Wearable plant sensors for real-time stress tracking [7]. (4) Metabolomic profiling for identifying stress biomarkers [8]. (5) Machine Learning (ML) algorithms to analyze the complex and multimodal datasets [9]. These advancements emphasize the importance and relevance of integrating smart sensing into contemporary plant health monitoring systems.

Electrical Impedance Spectroscopy (EIS) is a non-invasive and non-destructive approach used to measure the electrical impedance of plant tissues to evaluate their physiological state. By capturing frequency and phase-dependent impedance variations, EIS provides insights into key biological processes such as cell membrane integrity, ion transport, and water content. These changes often indicate stress responses, making EIS a valuable tool for the early diagnosis of plant stress [10,11]. When integrated with environmental sensors that monitor humidity, light, and temperature, EIS contributes to a comprehensive multimodal system for real-time plant health assessment under differing environmental conditions.

Machine learning techniques have revolutionized agricultural monitoring by enabling automated pattern recognition and predictive analytics using sensor data. These models have demonstrated high performance in both regression and classification tasks, supporting applications such as irrigation optimization, disease detection, and yield forecasting [6,9]. By analyzing complex multimodal datasets, ML techniques can uncover hidden patterns to provide timely insights into plant stress responses, thus enhancing decision-making and promoting resource-efficient, precision agriculture.

The study aims to develop an intelligent plant stress assessment framework to enable real-time, non-invasive detection and prediction of stress responses in plants. The primary goals of this study are:

1. To collect and analyze multimodal sensor data (temperature, humidity, and EIS) under various environmental stress conditions.
2. To identify and predict stress-induced physiological responses in plants using machine learning.
3. To propose a novel ensemble learning model for enhanced prediction accuracy.
4. To evaluate the efficacy of the proposed model against conventional regression techniques for plant stress prediction.

Several studies have explored plant responses to environmental stress using various sensing and ML approaches. Liew et al. (2008) demonstrated the use of thermal and fluorescence imaging for early pathogen detection and discussed spectral reflectance analysis and transgenic phytosensors for identifying nutrient and water stress [12]. Singh et al. (2020) reviewed plant responses to abiotic stressors—such as heat, salinity, and drought—highlighting the roles of secondary metabolites, phytohormones, and transcription factors in stress regulation [8]. Wang et al. (2021) examined plant physiological responses to temperature extremes and humidity [13].

Hamed et al. (2016) employed EIS to examine how the halophyte Cakile maritima adapts to salinity under different growth conditions, revealing that hydroponic systems induce higher stress levels and that impedance parameters vary dynamically with salt exposure [10]. Kashyap et al. (2021) further validated EIS as a non-destructive method to detect early physiological stress in plants [11]. Wearable sensors, as described by Lee et al. (2021), enable real-time monitoring of biomarkers and microenvironmental parameters in plants [7].

Machine learning has shown promise in agricultural diagnostics. Patel et al. used ML algorithms for early plant disease detection and improved crop management [9]. Unmanned Aerial Vehicle (UAV)-based multispectral imagery was applied by Zhang et al. (2019) to map maize water stress, leading to empirical models for the Crop Water Stress Index (CWSI) [6]. An ML model using k-mer-based feature encoding and Support Vector Machines (SVM) has been successfully applied by Pradhan et al. (2023) to predict abiotic stress–responsive microRNAs, highlighting the potential of computational tools in stress-resilient crop development [14]. The GenPhenML framework, combining molecular markers and phenotypic traits with ML by Akbari et al. (2024), demonstrated high accuracy (R^2^ > 0.99) in predicting and classifying barley genotypes under drought and salinity stress using neural networks [15].

Despite these advances, existing research often focuses on isolated stress factors, lacks integration across sensing modalities, or fails to support real-time, scalable monitoring. Most approaches rely on a single data type (e.g., imaging, EIS, or weather sensors), leaving the synergistic potential of multi-sensor fusion underutilized. The application of EIS with machine learning, especially across diverse plant species and environments, remains underexplored. To address these gaps, this study proposes a novel AI-driven framework that integrates EIS, temperature, and humidity data with ensemble learning techniques to enable early detection, accurate classification, and real-time prediction of plant stress, thereby supporting precision and sustainability in agriculture.

## 2. Materials and Methods

This section describes the experimental setup, including the plants monitored and the environmental conditions simulated. It details the data collection process and the machine learning algorithms applied for analysis.

**Figure 1:**
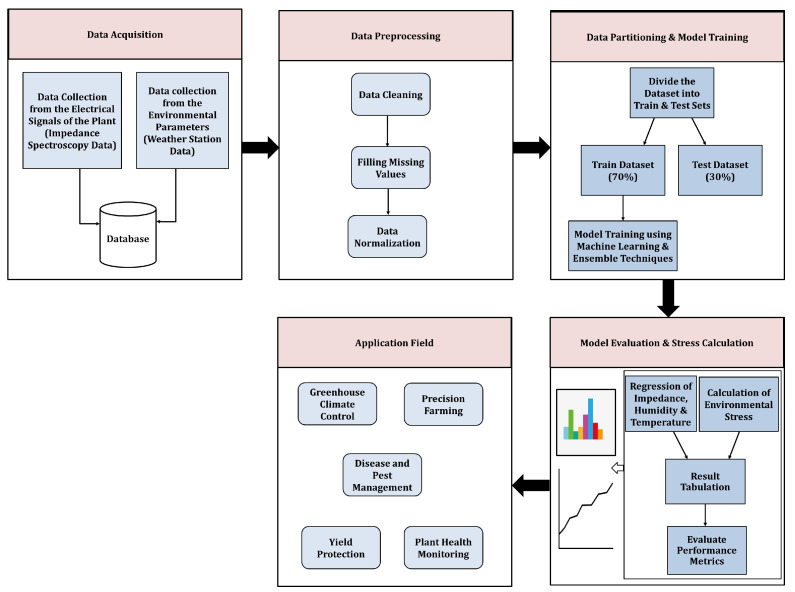
Proposed methodology and the application area of the proposed research.

Electrical impedance spectroscopy measurements were collected by Dr. Lee Bar-On at Tel-Aviv University for 30 to 60 days for each experiment involving various plants [16,17]. The experimental configuration is detailed below. Figure 1 illustrates both the methodology employed in the present research and the application domain of the proposed research.

### 2.1. Experimental Setup and Data Acquisition

**Figure 2:**
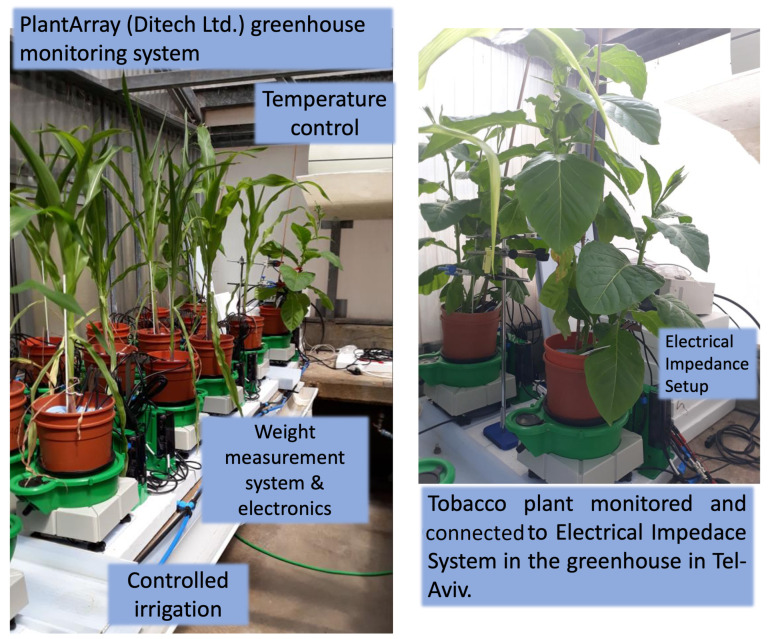
Experimental systems setup in greenhouse utilizing PlantArray system and monitored environment.

The data examined in this study were collected using a combination of methods to assess plant stress. This data was subsequently analyzed with various AI techniques for applications and predictions in precision agriculture. The experiments were conducted in an outdoor greenhouse facility, where a wide range of tests provided substantial data on plant responses and activities, measured through established plant physiological monitoring methods. Figure 2 demonstrates the experimental system setup in the greenhouse using the PlantArray^TM^ system (Plant-Ditech Ltd., Yavne, Israel) and a monitored environment, in addition to the collection of data simultaneously using the electrical impedance response across a variety of frequencies.

This comparative approach and study, which provides reliable data from multiple systems simultaneously over time, lays the foundation for developing and implementing various AI algorithms to predict plant stress.

1. Gravimetric System:
  An advanced gravimetric plant monitoring system was employed as the primary setup for automated whole-plant phenotyping. This system captured data related to plant weight and water use efficiency, leveraging the gravimetric method, which is widely recognized for its ability to reflect plant health and growth patterns [18,19]. Monitoring water usage further enabled insights into the physiological status and development of the plant.
2. Four-Point-Probe Electrical Impedance Spectroscopy System:
  Alongside the gravimetric system, novel four-point probe impedance spectroscopy measurements were conducted. These measurements continuously gathered data directly from the plant stem over periods ranging from weeks to two months. The setup, detailed and modeled in [16], used a four-point probe configuration to ensure accuracy and reliability. Impedance magnitude and phase values were collected spanning the frequency range of 50 Hz to 2 MHz, with samples taken every 9 min.
3. Environmental Monitoring in Greenhouse:
  The experimental work took place in a controlled greenhouse facility at Tel Aviv University, Israel. This environment, although located outdoors and naturally lit, was systematically regulated for temperature and humidity. The tobacco plant used in the study was connected to the impedance system and monitored throughout the experiment. Around 15 young tobacco plants, cultivated for 3–4 months, were used in the study. These plants had stem diameters ranging from 0.7 to 1.1 cm and an overall height of approximately 0.7 to 1 m. They were cultivated in 3.9 L pots containing coarse sand. The Vapor Pressure Deficit (VPD) within the greenhouse was continuously monitored and exhibited consistent patterns over multiple days. Each pot was irrigated daily during the nighttime hours (around 9PM) with 2 L of water to ensure saturation and promote healthy plant development. A centrally located weather station within the greenhouse continuously recorded vital environmental parameters such as plant weight, Relative Humidity (RH), temperature, Volumetric Water Content (VWC), and Vapor Pressure Deficit (VPD). These readings supported the identification of plant stress and optimization of growing conditions.

Table 1 provides comprehensive overview of experimental parameters gathered from three primary systems: the impedance spectroscopy setup, the gravimetric plant array system, and the environmental monitoring unit. Each parameter is described along with its corresponding unit and the range of values observed during the experiment. These parameters form the foundation of the gathered dataset and are essential for analyzing plant stress and physiological responses under varying environmental conditions. In addition to the detailed list of experimental parameters, a subset of the collected data is presented in Table 2 to illustrate the structure and variability within the sample dataset.

### 2.2. Data Pre-Processing and Model Training

#### 2.2.1. Data Analysis

**Figure 3:**
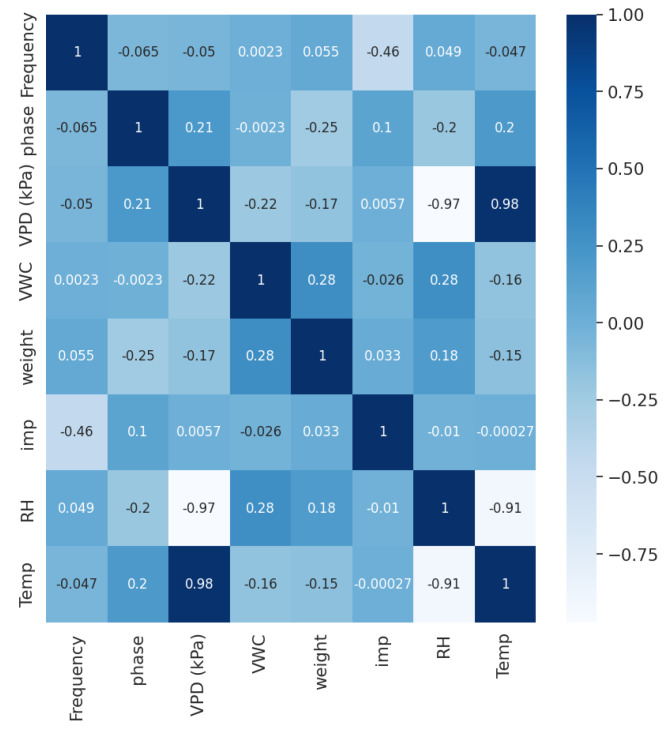
Heatmap for environmental stress parameters.

A heatmap is a graphical tool that employs colors to depict the relative values of each data point in a matrix, allowing for easy visualization of patterns and correlations among various parameters. In the heatmap, darker shades represent higher values, while lighter colors represent lower values. In this research, parameter correlation values were analyzed using a heatmap to investigate their relationships. The heatmap in Figure 3 displays the measured correlation matrix, showing a strong inverse relationship between RH & VPD, the strong direct relationship between temperature & VPD, and moderate relationship between frequency & impedance. The heatmap also served as a diagnostic tool to ensure that each independent variable had a meaningful relationship with the dependent variable while minimizing multicollinearity among the independent variables. This preliminary check helps in identifying and possibly discarding redundant or irrelevant features that may adversely affect model performance. However, in this study, all variables met the correlation criteria, and thus, no features were excluded from further analysis.

#### 2.2.2. Data Preparation

A total of 796,830 data entries from the collected dataset were included for modeling. The experimentally acquired data were cleaned and pre-processed using various techniques. The raw measurements were consolidated into a single file and processed as follows:

- Handling Missing Values:
  Missing entries were imputed using hot-deck imputation, which involves substituting null values with data from a similar entry within the dataset [20].
- Data Standardization:
  Each data sample was individually normalized to the unit norm, rescaling values to a common range, usually between 0 and 1.
- Dividing the Dataset into Train and Test Units:
  The dataset was split into 70% for training (557,781 instances) and 30% for testing (239,049 instances) to develop and assess the machine learning models.

### 2.3. Model Training

Training a machine learning model involves using data to learn and adjust its parameters. This step includes implementing an AI-based solution to predict required parameters, such as the impedance, relative humidity, and temperature, using the acquired data. These parameters will indicate the optimal conditions for plant growth. To ensure a comprehensive evaluation, several machine learning models were employed in this study. K-Nearest Neighbors (KNN) and Decision Tree (DT) were selected for their simplicity, interpretability, and effectiveness in modeling non-linear patterns. Multivariate Linear Regression (MLR) was included to provide a baseline for comparison with more sophisticated models. AdaBoost and the DT+AdaBoost combination were chosen to benefit from the boosting technique’s ability to reduce bias and variance, enhancing overall prediction performance. Additionally, a Multi-Layer Perceptron (MLP) was utilized to model intricate, non-linear relationships in data, leveraging the strength of neural network architectures. This diverse model selection helps to comprehensively assess performance across various algorithmic families. These models are described below.

- Decision Tree (DT):
  The Decision Tree (DT) regressor operates by iteratively dividing the input data into increasingly smaller subsets based on a set of splitting criteria until each subset consists of data points that share similar values of the target variable. The splitting criteria are chosen based on a measure of impurity or error, such as mean absolute or squared error. Once built, predictions for new data points are generated by navigating the tree from the root to the leaf node, where predicted output is the mean or median of training examples in that leaf node. Decision tree regressors can be prone to overfitting [21].
- K-Nearest Neighbors (KNN):
  KNN regression is a non-parametric algorithm in machine learning. It forecasts a new instance by considering k close neighbors and averaging their target values. The k parameter requires fine-tuning to avoid overfitting [22].
- Multivariate Linear Regression (MLR):
  In MLR, the model can be represented by a set of linear equations, one for each dependent variable. Multivariate linear regression aims to estimate the coefficients that reduce the difference between observed values and the model’s predicted values across all dependent variables. This is typically achieved using techniques such as least squares regression, which reduces the total sum of squared errors across all dependent variables [23]. The equation for MLR is:
  (1) $$\begin{array}{c} y_{1}=b_{10}+b_{11}x_{1}+b_{12}x_{2}+b_{13}x_{3}+b_{14}x_{4}+b_{15}x_{5}+\epsilon_{1}\\ y_{2}=b_{20}+b_{21}x_{1}+b_{22}x_{2}+b_{23}x_{3}+b_{24}x_{4}+b_{25}x_{5}+\epsilon_{2}\\ y_{3}=b_{30}+b_{31}x_{1}+b_{32}x_{2}+b_{33}x_{3}+b_{34}x_{4}+b_{35}x_{5}+\epsilon_{3} \end{array}$$
  where: $y_{1},y_{2},y_{3}$ denote the dependent variables and $x_{1},x_{2},x_{3},x_{4},x_{5}$ represent independent variables. $b_{ij}$ are the coefficients for the independent variables, $x_{j}$ in the equation for dependent variable $y_{i}$, $b_{i0}$ are the intercepts for each dependent variable and $\epsilon_{i}$ represents the error terms for each dependent variable.
- AdaBoost:
  The AdaBoost regressor is an adaptive method that adjusts the weights of individual models to enhance overall system performance. It combines several weak learning models to construct a robust and precise model. In every iteration, a weak learning model is trained using a subset of the training data, and its efficacy is assessed. Instances misclassified by the weak model are assigned higher weights, and this process repeats until the desired level of accuracy is obtained. The final prediction is the weighted mean of all weak model predictions [24].
- Multi-Layer Perceptron (MLP):
  An MLP regressor comprises several layers of nodes (neurons) connected by weighted edges, where every node in one layer is connected to each node in adjacent layers. It processes input values through a series of hidden layers, each applying an activation function to the weighted sum of its inputs [21].

The value of tuning parameters for machine learning models employed in this current study is tabulated in Table 3.

#### 2.3.1. Proposed Boosting-Based Ensemble Approach: AdapTree (Adaptive Boosted Tree for Plant Stress Analysis)

Boosting is an advanced ensemble technique that focuses on improving the performance of weak learners by combining them into a robust predictive model. The boosting process entails training models in sequence, with every subsequent model addressing errors made by its predecessor. A stepwise outline of the boosting-based ensemble approach is presented here:

 Step 1: Create the base model. The first step is to define the base model. The base model serves as the weak learner in the ensemble. The choice of the base learner allows for capturing non-linear relationships and interactions between variables.
 Step 2: Initialize weights and train the first model. Initialize weights for all training samples equally. Train the base model on the weighted training dataset. The initial model will be focused on minimizing errors across the dataset.
 Step 3: Compute model errors and update weights. After training the first base model, compute the errors for each training instance. Increase the weights of incorrectly predicted samples so that the next model focuses more on these challenging cases. Update the weights accordingly to reflect the importance of each sample.
 Step 4: Train subsequent models. Train additional base models in sequence, where every model focuses on the errors of the predecessor model. Each new model is trained on a modified dataset with updated weights to emphasize the mistakes made by earlier models.
 Step 5: Aggregate models using boosting algorithm. Combine the predictions from all trained base models using the boosting algorithm. The boosting algorithm assigns a weight to each model based on its accuracy, and the final prediction is computed as a weighted sum of individual model predictions from all models.
 Step 6: Make the final prediction. Use the aggregated predictions to make the final prediction. The combined output from the boosting algorithm will result from the weighted majority vote or the weighted sum of the predictions made by all the base learners.

Assuming *T* total base models, the final prediction $\hat{y}$ for new data point *x* is calculated as:

(2) $$\hat{y}\left(x\right)=\sum_{t=1}^{T}\alpha_{t}\cdot h_{t}\left(x\right)$$

where: $\hat{y}\left(x\right)$ denotes final prediction for input *x*, *T* denotes number of base models, $\alpha_{t}$ denotes weight assigned to *t*-th model, determined by boosting algorithm, and $h_{t}\left(x\right)$ denotes prediction of *t*-th base model for input *x*.

In the current study, the proposed AdapTree model integrates decision trees with the AdaBoost ensemble method. Decision trees were selected for their interpretability and capability to handle non-linear relationships, which are common in biological data such as plant stress parameters. However, single decision trees may suffer from instability and overfitting. To overcome these limitations, AdaBoost was incorporated, as it improves model robustness by sequentially combining multiple weak learners and focusing on difficult-to-classify instances. This ensemble approach enhances generalization and predictive accuracy. The integration of these methods was particularly suitable for the plant stress prediction task, where subtle variations in environmental and physiological parameters require a model that can adaptively learn from complex patterns while reducing bias and variance.

Thus, for the given case, DT is the base learner, and AdaBoost is the boosting method. The prediction of the decision tree model ($h_{1}$) is given by $h_{1}\left(x\right)$. $h_{2}\left(x\right)$ is derived from the boosting process where $h_{2}$ represents the aggregated model based on AdaBoost. The formula for the two models would be:

(3) $$\hat{y}\left(x\right)=\alpha_{1}\cdot h_{1}\left(x\right)+\alpha_{2}\cdot h_{2}\left(x\right)$$

where: $\alpha_{1}$ is the weight for the DT model, $\alpha_{2}$ is the weight for the AdaBoost model, $h_{1}\left(x\right)$ is the prediction from the DT, $h_{2}\left(x\right)$ is the prediction from the AdaBoost model, $\alpha_{1}\cdot h_{1}\left(x\right)$ represents the contribution of the DT model and $\alpha_{2}\cdot h_{2}\left(x\right)$ represents the contribution of the AdaBoost model. The weights $\alpha_{1}$ and $\alpha_{2}$ are determined during the boosting process, reflecting the performance of each model.

**Figure 4:**
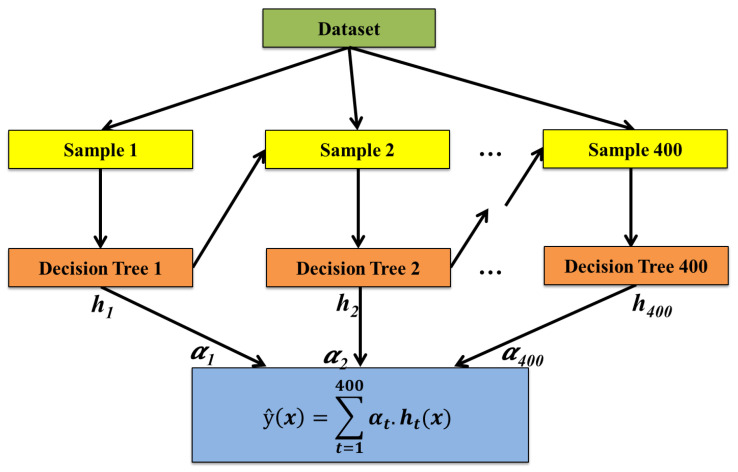
Diagram illustrating the proposed boosting algorithm AdapTree.

Figure 4 illustrates the working procedure of this approach.

#### 2.3.2. Plant Stress Calculation

Plant stress can be computed using the Environmental Stress Index (ESI) measure, which combines various environmental factors and physiological responses. A general formula for ESI includes weighted contributions from each factor.

(4) $$\mathrm{ESI}=\left(\frac{T-T_{\mathrm{opt}}}{T_{\max}-T_{\min}}\right)\cdot w_{1}+\left(\frac{\mathrm{RH}-{\mathrm{RH}}_{\mathrm{opt}}}{{\mathrm{RH}}_{\max}-{\mathrm{RH}}_{\min}}\right)\cdot w_{2}+\left(\frac{\mathrm{PI}-PI_{\mathrm{opt}}}{{\mathrm{PI}}_{\max}-{\mathrm{PI}}_{\min}}\right)\cdot w_{3}$$

where the acronyms mean the following: *T*: Temperature; RH: Relative Humidity; PI: Plant Impedance; $w_{1}$, $w_{2}$, $w_{3}$: The weights assigned to each factor based on their importance, opt, max, min: Optimal, maximum, and minimum values for each factor that the plant can tolerate before experiencing stress.

The weights $w_{1}$, $w_{2}$, and $w_{3}$ reflect the relative importance of each factor (T, RH, and PI) in determining the overall environmental stress on the plant. The significance of these factors may differ based on the plant species and environmental conditions. The current study assigns equal weight to each factor (T, RH, and PI). Thus, each factor would weigh:

(5) $$w_{1}=w_{2}=w_{3}=\frac{1}{3}$$

## 3. Results and Discussion

This section presents the findings of the study, including the performance of machine learning models used to predict plant stress and the effectiveness of these models in identifying stress conditions (described in Table 4, Table 5 and Table 6). It discusses the implications of these findings for plant health monitoring and stress mitigation. The efficacy of the trained models is assessed using various regression performance metrics.

1. Mean Absolute Error (MAE):
  The *MAE* represents average absolute differences between predicted and actual values.
  (6) $$MAE=\frac{1}{n}\sum (|y_{i}-\hat{y_{i}}\left|\right)$$
  where *n* stands for the total number of observations, $y_{i}$ denotes the actual value of the output variable, and $\hat{y_{i}}$ denotes the predicted value of the output variable.
2. Mean Squared Error (MSE):
  The *MSE* computes the average of squared differences between predicted and actual values.
  (7) $$MSE=\frac{1}{n}\sum {\left(y_{i}-\hat{y_{i}}\right)}^{2}$$
3. Root Mean Squared Error (RMSE):
  The *RMSE* represents the square root of the *MSE* and quantifies the standard deviation of the differences between predicted and actual values.
  (8) $$RMSE=\sqrt{\frac{1}{n}\sum {\left(y_{i}-\hat{y_{i}}\right)}^{2}}$$
4. Pearson Correlation Coefficient (PCC):
  The *PCC* calculates the covariance between two variables, measuring how much the variables vary together. The value of the *PCC* ranges between −1 and +1. It is calculated using the formula:
  (9) $$PCC=\frac{\sum (\left(X-\bar{X}\right)\left(Y-\bar{Y}\right))}{\left(n-1\right)\cdot S_{x}\cdot S_{y}}$$
  where: *X* and *Y* represent the two variables being analyzed, $\bar{X}$ and $\bar{Y}$ denote their respective means, *n* represents the total number of observations, and $S_{x}$ and $S_{y}$ represent the standard deviations of *X* and *Y*, respectively. When the PCC approaches 1, it signifies a strong positive linear correlation between the variables, suggesting that as one variable rises, the other typically increases too. Conversely, a PCC close to –1 represents a strong negative relationship, meaning that as one variable increases, other typically decreases.
5. *R*-Square ($R^{2}$):
  The *R*-squared measures the proportion of variability in the output variable explained by the input variables in the model.
  (10) $$R^{2}=1-\frac{SS_{res}}{SS_{tot}}$$
  where: $SS_{res}$ represents the sum of squared residuals, representing the sum of squared differences between actual and predicted values, and $SS_{tot}$ represents the total sum of squares, representing the sum of squared differences between actual values and mean of the output variable. A higher R-squared indicates better model performance.

The results depicted in Table 4, Table 5 and Table 6 illustrate the effectiveness of the proposed AdapTree model in predicting plant stress parameters using impedance, humidity, and temperature data, respectively. The performance of this model was compared against MLP, MLR, DT, AdaBoost, and KNN.

Table 4 presents the efficacy of various regression models in impedance parameter prediction. The proposed AdapTree model achieved the lowest MAE (22.789), MSE (19,812.34), and RMSE (134.565), indicating its superior accuracy in estimating impedance changes due to plant stress. Furthermore, the model demonstrated the highest R^2^ (0.993125) and PCC (0.996564), suggesting a strong relationship between predicted and actual impedance values. In contrast, traditional regression models such as MLR performed poorly, with higher error values (MAE: 1523.617, RMSE: 1725.631) and a lower R^2^ (0.224172), indicating its limitations in capturing complex stress-related impedance variations. AdaBoost and MLP showed moderate performance, while DT and KNN exhibited relatively competitive results but fell short of the proposed AdapTree.

Table 4 suggests that a high error can occur even when $R^{2}$ is high. However, as noted by [25], metrics do not have absolute numeric values and require a benchmark for meaningful interpretation. For instance, the simplest model that minimizes the root squared error predicts the standard deviation for all samples. For the MAE, the simplest model predicts the median. The standard deviation of the sample is 1961.246, and the RMSE is 134.565. The median of the sample is 1994.046, and the MAE is 22.789. The values indicate that both the RMSE and MAE are relatively low compared to the benchmark values. 

Table 5 compares the models’ performance in predicting humidity variations under environmental stress conditions. The proposed AdapTree model again outperformed all baseline models, achieving the lowest MAE (1.51 × $10^{-5}$), MSE (4.85 × $10^{-5}$), and RMSE (0.006966), with an R^2^ and PCC of 0.999999. The MLR model performed reasonably well with an R^2^ of 0.948011 but showed slightly higher errors (MAE: 4.064481, RMSE: 5.018066). MLP performed the weakest, with an R^2^ value of 0.629311, indicating its limited ability to model humidity-dependent stress responses. The results suggest that KNN, tree-based architectures like DT, the ensemble-based methods like AdaBoost, and the proposed method provide better generalization compared to other regression techniques. 

For temperature prediction, shown in Table 6, the proposed AdapTree model achieved the best predictive accuracy, with an R^2^ of 0.999998 and PCC of 0.9999992. The model yielded the lowest MAE (2.51 × $10^{-5}$), MSE (2.51 × $10^{-5}$), and RMSE (0.0050099), outperforming all other models. The DT exhibited similar R^2^ as the proposed model, relatively competitive results, but obtained comparatively higher error (MSE and MAE: 3.76 × $10^{-5}$, RMSE: 0.006136) and lower PCC (0.99999). AdaBoost (R^2^: 0.989251) and KNN (R^2^: 0.987155) displayed suboptimal results, likely due to overfitting and sensitivity to noise. Though MLR lagged behind the proposed approach, its performance was good (R^2^: 0.965393, MAE: 0.62162), affirming the strength of linear models in capturing the nonlinear interactions between temperature and plant stress responses. The lower performance of MLP (R^2^: 0.505675, MAE: 2.044328) justifies that it suffers from the problem of vanishing gradient during training, hindering convergence.

The experimental findings indicate that the proposed AdapTree model consistently outperforms other machine learning and regression models across all three environmental parameters. Its superior predictive capability stems from the boosting mechanism, which refines decision trees iteratively, minimizing errors and improving model robustness. The MLP regression model failed to capture the nonlinear relationships inherent in plant stress responses, while DT, though competitive, exhibited slightly higher error rates. 

The predictive capability of the AdapTree model is also highly dependent on the quality of input data acquired through environmental sensors. As stated earlier, sensors were used in this study to continuously monitor key stress indicators such as temperature, humidity, and impedance. These real-time readings form the foundational input for the machine learning model, enabling it to detect subtle changes in plant responses under varying environmental conditions. The integration of sensor-based monitoring enhances the timeliness and accuracy of the predictions, making the model more effective for dynamic agricultural environments. Thus, the performance of the AdapTree model is strongly influenced by the accuracy and reliability of the sensor data. On the contrary, noisy, biased, or missing readings can adversely affect model training and inference. To address this, preprocessing techniques such as filtering and normalization were applied to ensure high-quality input data. 

These findings underscore the practical viability of the AdapTree model for precision agriculture, where early and accurate detection of plant stress is critical for timely intervention. By leveraging real-time, sensor-based data and a robust ensemble learning framework, the model offers a scalable solution for adaptive crop management. Compared to existing methods, which may either overlook subtle physiological changes or lack generalizability across variable conditions, AdapTree demonstrates a superior ability to capture complex environmental interactions. This advancement not only contributes to improved yield forecasting and resource optimization but also paves the way for automated, AI-driven agricultural decision-support systems. 

Future research may focus on optimizing sensor placement, improving sensor hardware, or incorporating signal processing algorithms to further enhance the quality of data acquisition. Such improvements would not only increase the robustness of the AdapTree model but also advance its practical deployment in real-world agricultural settings.

### Comparison with the Baseline Studies

Table 7 compares the results between the proposed ensemble and the existing state-of-the-art, thus highlighting its effectiveness in predicting critical environmental factors for plant growth.

As shown in Table 7, various artificial intelligence models have been employed across different studies for plant stress detection, targeting various stress parameters and plant types. Traditional machine learning models like SVM achieved moderate accuracy (ranging from 80.38% to 84.57%) in detecting multiple stress types (cold, drought, heat and salt) [14], while deep learning and ensemble approaches such as neural networks (R^2^: 0.9999), GenPhenML (R^2^: 0.9995) [15], GoogLeNet (accuracy: 92.9% and 97.9%) [27], and extreme gradient boosting (R^2^: 0.99) [29] have demonstrated improved performance across specific stress conditions. The ensemble KNN used by Azrai et al. (2024) achieved a stress tolerance index of 0.82 for drought stress in maize plants [26]. Sharma et al. (2024) reported moderate regression results using a deep learning model H_2_O-3 (R^2^: 0.80) for water stress in wheat plants [28]. 

Notably, the proposed AdapTree model outperforms all existing approaches, achieving an exceptionally high R^2^ value with minimal prediction errors across all three stress parameters (MAE: 22.789, RMSE: 134.565 and R^2^: 0.993125 for impedance; MAE: 1.51 × $10^{-5}$, RMSE: 0.006966 and R^2^: 0.999999 for RH and MAE: 2.51 × $10^{-5}$, RMSE: 0.0050099 and R^2^: 0.999998 for temperature). This reflects its superior ability to model complex interactions among impedance, humidity, and temperature parameters in tobacco plants. Compared to other studies that often focus on single stress types or limited input features, the proposed work integrates multi-parametric sensing with a robust ensemble learning model, making it a more comprehensive and accurate solution for real-time plant stress monitoring. 

These findings highlight the capability of ensemble learning approaches in plant stress monitoring and suggest that the integration of boosting-based models can significantly enhance predictive accuracy for agricultural applications. By leveraging advanced machine learning techniques, this study contributes to real-time plant health assessment, enabling more proactive agricultural management strategies. 

Although ensemble learning methods and sensor-based monitoring have been individually applied in agricultural contexts, the integration of a boosting-based ensemble model—AdapTree, combining AdaBoost with decision trees—with electrical impedance spectroscopy and real-time environmental sensing (temperature and humidity) represents a novel methodological contribution. To the best of our knowledge, this is the first study to fuse these three modalities into a unified, data-driven framework for plant stress prediction. Unlike previous models that typically rely on single-modality inputs or general-purpose datasets, this approach leverages the synergistic strengths of impedance-based physiological sensing and adaptive ensemble learning to enhance prediction accuracy and robustness. This integrated method addresses key limitations in prior research, such as delayed stress detection, low model generalizability, or the absence of continuous sensing mechanisms, and demonstrates superior performance with R² scores exceeding 0.99 across all parameters. 

It is important to highlight that, although the AdapTree model demonstrated superior performance across multiple evaluation metrics—including MAE, MSE, RMSE, R^2^, and PCC—for predicting impedance, humidity, and temperature under environmental stress conditions, it is important to acknowledge its limitations. Firstly, AdapTree, being an ensemble of decision trees boosted via AdaBoost, is inherently more complex, which can make the interpretation of individual feature contributions less transparent compared to simpler models. Although correlation analysis was employed, it only provides a high-level view of variable relationships. More advanced model explainability techniques could be explored in future work to gain deeper insights into feature importance and decision-making within the ensemble. 

Additionally, AdaBoost can be sensitive to noisy data and outliers, potentially affecting model robustness in less curated datasets. The computational overhead associated with ensemble learning could also limit the model’s deployment in real-time or resource-constrained agricultural settings. Moreover, while the results are promising, the dataset used in this study is restricted to the Tobacco plant, which may limit the generalizability of the model to other plant species or varying environmental conditions. Future research could incorporate more diverse datasets to validate the scalability and adaptability of the AdapTree model across different crop types and growth environments.

## 4. Conclusions

Based on the research analysis, the proposed boosting-based ensemble approach combining AdaBoost and decision trees (AdapTree) outperforms other regression models for predicting plant stress using electrical impedance spectroscopy and environmental data. The study achieved an R^2^ score of 0.993 for estimating impedance magnitude and 0.999 for both relative humidity and temperature, demonstrating the model’s robustness and accuracy across key physiological parameters.These results validate the effectiveness of ensemble learning in capturing nonlinear plant responses and underscore the value of integrating real-time sensor data into predictive modeling. The findings support the potential of AdapTree to contribute to precision agriculture by enabling timely, accurate monitoring of plant health, which in turn can improve resource use and crop management strategies. While the current study was conducted under controlled greenhouse conditions to ensure consistency and minimize environmental variability, it is acknowledged that such settings do not fully capture the complexity of real-world agricultural environments. 

For future work, the study can be expanded by incorporating additional physiological indicators such as light intensity and chlorophyll content to further refine stress prediction. Moreover, addressing practical deployment challenges—including sensor scalability, species variability, and real-time data processing—will be essential for field adoption. Investigating techniques such as signal compression, edge/cloud integration, and adaptive sensor placement strategies could further enhance the system’s applicability in real-world, data-driven agricultural decision-making. Also, to fully capture the complexity of real-world agricultural environments, future work can extend the current experiments to field conditions with varying climatic, soil, and pest-related challenges. This will help validate the robustness and adaptability of the proposed system under practical scenarios. 

## Figures and Tables

**Table 1:** List of gathered parameters from the experimental systems with their description, unit, and range.

Experimental Systems	Gathered Parameter	Description	Units	Range
Impedance Spectroscopy Setup	Frequency	The frequency value at which measurements were collected.	Hertz (Hz)	50 to 3,940,000
	Impedance Magnitude (Impedance)	Ratio between the Alternating Current (AC) voltage to the AC phasors.	Ohm (Ω)	282.3103 to 7958.325
	Impedance Phase (Phase)	The delay of the angular component of a periodic wave vs. that of the excitation.	Degree (°)	−40.232 to −3.127
Gravimetric Plant Array System	Plant Weight	The weight of the plant.	Kilograms (kg)	0.54347 to 1.14102
	Volumetric Water Content (VWC)	The amount of water present in a given volume of soil.	Percentage (%)	0.02961 to 0.18781
Environmental Parameters	Relative Humidity (RH)	The proportion of water vapor present in air to the maximum amount the air can hold.	Percentage (%)	27.5 to 91.8
	Temperature	The temperature at which the plant is maintained.	Degree Celsius (°C)	19 to 30
	Vapor Pressure Deficit (VPD)	The difference between the moisture content in the air and the maximum amount of moisture the air can hold.	Kilopascal (kPa)	0.19159 to 3.07549

**Table 2:** Sample dataset.

Explanatory Variables					Outcome Variables		
Frequency	Phase	VPD	VWC	Weight	Impedance	RH	Temperature
1,000,000	−20.541	0.44159	0.18251	1.01719	327	81.1	20
101	−3.563	0.29206	0.18429	1.01821	4529.482	87.5	20
1010	−16.043	0.28972	0.1835	1.0172	3892.253	87.6	20
10,100	−32.47	0.32935	0.18468	1.01668	1869.21	85	19
101,000	−34.239	0.54206	0.18291	1.01812	785.5841	76.8	20
1020	−15.933	0.34252	0.18311	1.01747	3821.377	84.4	19
10,200	−32.441	0.25234	0.18291	1.01771	1846.688	89.2	20
102,000	−34.205	0.24767	0.18311	1.01713	775.136	89.4	20

**Table 3:** List of machine learning models with their tuned parameters.

S.No.	Model	Tuning Parameters
1	DT	criterion = ‘gini’
2	KNN	n_neighbors = 2
3	MLR	fit_intercept = True; n_jobs = −1; max_iter = 1000; tol = 0.0001
4	AdaBoost	n_estimators = 100
5	MLP	hidden_layer_sizes = (20, 30); max_iter = 200; alpha = 0.001; solver = ‘adam’

**Table 4:** Comparison of regression models for impedance parameter of environmental stress.

Regression Models	R-Square	MSE	RMSE	MAE	Pearson Coefficient
MLP	0.957907	161,561.5	401.9472	288.1211	0.978876
MLR	0.224172	2,977,801	1725.631	1523.617	0.473478
DT	0.981457	26,471.97	162.7021	33.40362	0.992099
AdaBoost	0.936375	245,359.1	495.3374	347.8731	0.96841
KNN	0.982715	66,658.18	258.1824	114.5485	0.991329
Proposed	0.993125	19,812.34	134.565	22.789	0.996564

**Table 5:** Comparison of regression models for humidity parameter of environmental stress.

Regression Models	R-Square	MSE	RMSE	MAE	Pearson Coefficient
MLP	0.629311	179.5437	13.39939	8.703461	0.79441
MLR	0.948011	25.18098	5.018066	4.064481	0.973659
DT	0.996941	0.00031	0.017612	8.24 × 10^−5^	0.998189
AdaBoost	0.995829	2.020256	1.421357	1.13163	0.997971
KNN	0.993734	3.035085	1.74215	1.221362	0.996878
Proposed	0.999999	4.85 × 10^−5^	0.006966	1.51 × 10^−5^	0.999999

**Table 6:** Comparison of regression models for temperature parameter of environmental stress.

Regression Models	R-Square	MSE	RMSE	MAE	Pearson Coefficient
MLP	0.505675	7.939475	2.817707	2.044328	0.711635
MLR	0.965393	0.555825	0.745537	0.62162	0.982544
DT	0.999998	3.76 × 10^−5^	0.006136	3.76 × 10^−5^	0.99999
AdaBoost	0.989251	0.172641	0.415501	0.382264	0.994867
KNN	0.987155	0.206302	0.454205	0.26097	0.993566
Proposed	0.999998	2.51 × 10^−5^	0.0050099	2.51 × 10^−5^	0.9999992

**Table 7:** Comparison of the proposed work with existing studies on plant stress detection using various ai models.

Author Name (Year)	Stress Parameter	Plant Type	Models Employed	Results Achieved
Pradhan et al. (2023) [14]	Cold Drought Heat Salt	-	SVM	Cold Accuracy: 84.57% Drought Accuracy: 80.62% Heat Accuracy: 80.38% Salt Accuracy: 82.78%
Akbari et al. (2024) [15]	Drought	Barley	Neural Network	MAE: 0.0727 RMSE: 0.0105 R^2^: 0.9999
Akbari et al. (2024) [15]	Salinity	Barley	GenPhenML	MAE: 0.1206 RMSE: 0.0308 R^2^: 0.9995
Azrai et al. (2024) [26]	Drought	Maize	Ensemble KNN	Stress Tolerance Index: 0.82
Chandel et al. (2024) [27]	Water	Maize Wheat	GoogLeNet	Maize Accuracy: 97.9% Wheat Accuracy: 92.9%
Sharma et al. (2024) [28]	Water	Wheat	H_2_O-3 Deep Learning Model	R^2^: 0.80
Singh et al. (2025) [29]	Biotic (Wilt Disease)	Chickpea	Extreme Gradient Boosting	R^2^: 0.99 RMSE: 0.72
Proposed	Impedance	Tobacco	AdapTree	MAE: 22.789 RMSE: 134.565 R^2^: 0.993125
	Humidity			MAE: 1.51 × $10^{-5}$ RMSE: 0.006966 R^2^: 0.999999
	Temperature			MAE: 2.51 × $10^{-5}$ RMSE: 0.0050099 R^2^: 0.999998

## Data Availability

The data will be made available on reasonable request.

## Abbreviations

The following abbreviations are used in this manuscript:

AI	Artificial Intelligence
ML	Machine Learning
EIS	Electrical Impedance Spectroscopy
UAV	Unmanned Aerial Vehicle
CWSI	Crop Water Stress Index
SVM	Support Vector Machines
VPD	Vapor Pressure Deficit
RH	Relative Humidity
VWC	Volumetric Water Content
AC	Alternating Current
KNN	K-Nearest Neighbors
DT	Decision Tree
MLR	Multivariate Linear Regression
MLP	Multi-Layer Perceptron
AdapTree	Adaptive Boosted Tree for Plant Stress Analysis
ESI	Environmental Stress Index
T	Temperature
PI	Plant Impedance
MAE	Mean Absolute Error
MSE	Mean Squared Error
RMSE	Root Mean Squared Error
PCC	Pearson Correlation Coefficient
R^2^	R-Squared
